# Fluorescent bionanosensor for picomolar detection of biomarkers in fluids

**DOI:** 10.1007/s00249-026-01825-8

**Published:** 2026-03-16

**Authors:** Aleksei Pashchenko, Marie Pospíšilová, Johana Šternová, Leontýna Varvařovská, Romana Široká, Hana Kalábová, Taťána Jarošíková, Šarka Beková, Sara Cruciani, Alois Nečas, Petr Novotný, Jiří Rulc, Gracian Tejral, Bruno Sopko, Margherita Maioli, Evzen Amler

**Affiliations:** 1 Second Faculty of Medicine, Charles University, Prague, 15006 Czech Republic; 2 Department of Biomedical Sciences, University of Sassari, Sassari, 07100 Italy; 3 Faculty of Biomedical Engineering, Czech Technical University in Prague, Kladno, 27201 Czech Republic; 4 University Centre for Energy Efficient Buildings, Czech Technical University in Prague, Trinecka , 1024, 27343 Bustehrad, Czech Republic; 5 Faculty of Veterinary Medicine, University of Veterinary and Sciences Brno, Brno, 61242, Czech Republic; 6 Student Science S.R.O, Prague, 19012 , Czech Republic; 7 ProNanoTech S.R.O, Všelibice, 46348, Czech Republic; 8 EBAS Nano S.R.O, Prague, 19012, Czech Republic

**Keywords:** Fluorescence bionanosensors, Polyacrylonitrile nanofibers, Early disease detection, Ultrasensitivity, Biotin-FITC, Functionalized nanomembranes

## Abstract

Fast and simple detection of diseases at their early stage and clinical monitoring of healing progress directly from body fluids, namely from urine, is a hot topic in modern personalized medicine. Functionalized nanofibers seem to be an ideal solution for the construction of specific and highly selective detection systems and microfluidic tools due to their extremely high ratio between their surface and volume, and also tunable sensitivity. The aim of this study is development of biosensors based on functionalized nanofibers leading to signal enhancement which would enable fluorescence detection and improve diagnostic accuracy in human fluids. Functionalized-PolyAcrylNnitrile (fPAN) membranes were fabricated and analyzed via SEM and fluorescence spectroscopy. Biotin-FITC binding was tested using a routine filtration setup and quantified with a Fluoromax4 spectrometer. Avidin-functionalized membranes showed strong, specific binding at picomolar levels Since the collected urine can be easily collected at volumes of more than 100 mL, and fluorescence measurement performed at a significantly smaller volume than 1 mL, our nanofiber-based system serves as an efficient specific enhancer and increases, thus, the detection limit by more than two orders of magnitude. This is already sufficient, for example, for a reliable fluorescence detection of urine markers in subpicomolar concentrations. Thus, this study describes a novel detection approach and application of fluorescence bionanosensors based on specifically functionalized polyacrylonitrile nanofibers for monitoring of subpicomolar concentrated markers in human fluids. These concentrations allow already monitoring of serious diseases at their early stage, which is crucial for distant and precision medicine.

## Background

Early-stage disease diagnosis plays a critical role in improving patient outcomes and survival rates by enabling timely intervention and treatment. The ability to detect disease at an early stage often depends on the identification of specific diagnostic biomarkers, which are biological molecules or physical parameters, indicative of various characteristics and related diseases. According to BEST (Biomarkers, EndpointS, and other Tools) classification, biomarkers can be divided by the following functions/disease characteristics: presence, progression, susceptibility/risk, diagnostic, monitoring, prognostic, predictive, pharmacodynamic/response, and safety (Silver Spring (MD) 2016: FDA, National Institutes of Health (US), 2016). Fluidic biomarkers can be represented in analyte by proteins, nucleic acids, or metabolites, and their detection allows for precise, targeted, and even personalized treatment approaches. However, detecting low concentrations of biomarkers faces several challenges, particularly in determining them from the background noise of other biological molecules. This requires highly sensitive and selective methods to measure small amounts of biomarkers in complex biological samples precisely.

The primary objective of this study was to explore the development and application of optical electrospun nanofibers-based fluorescence bionanosensors for the detection of standard and low concentrations of diagnostic biomarkers of early-stage diseases. The research aims to evaluate the performance of these sensors in terms of sensitivity, specificity, potential clinical applications, and usage after storage. By investigating the efficacy of these sensors, the study seeks to contribute to advancing early disease diagnosis and improving patient care outcomes.

Electrospun-produced nanofibers, known for their high surface area, biocompatibility, and tunable porosity, serve as an optimal platform for biosensing applications. Polyacrylonitrile (PAN)-based nanofibers have shown great promise in advancing filtration technology and chemical modification due to their unique properties. These properties include low density, thermal stability, high strength, and modulus of elasticity, making PAN an essential polymer in various medical applications. The combination of their specific properties and the ability to custom-modify their surfaces makes them a flexible platform for creating tailor-made filters for multiple applications in liquid processing and medical diagnostics (Bajwa et al. 2023; Fasiku (Oluwaseun) et al. 2020; Park et al.^2010^, Nalesso and Claudio 2017). PAN-based nanofibers have gained recognition for their flexibility and durability, making them suitable for ultrasensitive water, humid air filters, and various biomedical applications. The ability of PAN nanofibers to function effectively as bacterial filters stems from their robust physical characteristics, such as mechanical and thermal stability, water insolubility, and adaptability (Danagody et al. 2024). These attributes enable the development of filters that can resist various environmental conditions and provide consistent performance in trapping and removing bacteria from water and air sources, as well as the detection of biomarkers from human body fluids. Previous studies have demonstrated the feasibility of using PAN nanofibers in creating high-performance bacterial filters, which can be tuned for specific applications based on their area density and surface properties (Pashchenko 2023; Huang et al. 2024; Varvařovská et al. 2024; Varvarovska et al. 2024; Varvařovská et al. 2023). These studies highlight the importance of controlling nanofiber morphology, specifically size and distribution, to optimize filter capture efficiency. The unique properties of PAN nanofibers make them an excellent material for the manufacture of tunable bacterial filters, which are critical for the rapid removal and monitoring of bacterial contamination in liquids. Future research may continue to explore novel approaches to optimize PAN nanofiber-based filters and their integration into existing filtration systems, including bacterial sensing, up to hemodialysis applications (Danagody et al. 2024, Varvarovská et al. 2024).

Optical fluorescence bionanosensors offer a promising solution to these issues due to their potential for ultrasensitive detection. These sensors employ optical fluorescence techniques to identify and quantify biomarkers with high sensitivity and specificity. Their nanoscale size and functionalization capabilities allow targeted binding to specific biomarkers, making them ideal for early-stage disease detection. Furthermore, optical fluorescence bionanosensors can be integrated into portable, low-cost, multi-markers diagnostic devices, potentially revolutionizing point-of-care testing.

In clinical diagnostics, biomarker concentrations in body fluids are commonly used to assess disease presence and progression. For example, prostate-specific antigen (PSA) is a widely recognized biomarker for prostate cancer screening. PSA in blood is assumed to be a suitable screening indicator for additional prostate examination in men after 45 years. Our research takes into consideration the established cut-off of urinary PSA, and aimed at the established marker concentration for the biosensor preparation. The studies identified a urinary PSA cutoff at 150 ng/mL, with a sensitivity of 92.5% and specificity of 36% in distinguishing prostate cancer from benign prostatic hyperplasia (BPH) or normal prostate conditions. The receiver operating characteristic (ROC) curve and the area under the curve (AUC) have been instrumental in defining clinically relevant cutoffs (Bolduc et al. 2007). This highlights the importance of setting precise detection thresholds for accurate diagnostics. In the context of serum analysis, additional challenges must be addressed, including lower PSA concentrations, the presence of interfering compounds, and the need for more complex separation methods, including pre-treatment, or physical preparation, e.g., pre-filtration to eliminate larger compounds/centrifugal separation, etc. These issues are particularly important for biosensor development, especially in point-of-care applications. From the example of broad integrated PSA screening strategies we can learn the importance of description reference levels of biomarkers for different geographies, healthcare systems, and even differences in groups, such as age-related differences (age correction index), risk factors, and genetic predisposition. Results could be influenced by many external and internal factors that should be taken into account. Serum PSA level could be significantly influenced by test interpretation; for instance, using a 2.5 μg/L threshold increases the risk of false positives without improving cancer detection compared to a 4.0 μg/L cutoff. Screening typically targets men aged 40–74, with thresholds such as PSA > 4.0 μg/L, PSAV > 0.35 μg/L/year, or values above the 95th age-specific percentile. Median PSA levels range from 0.7 to 2.0 μg/L, with the 95th percentile reaching up to 6.5 μg/L. These values highlight the need for highly processed descriptions of already known and novel biomarkers, which sensitive biosensors are capable of reliably detecting in the low nanomolar or even picomolar range. That group of factors could be the additional reason for aiming our membrane functional concentration in this range, and the idea of applying developed systems as sample concentrators for standard markers. Effective integration of such tools into clinical workflows could improve early detection while supporting personalized screening strategies (Prostate-Specific Antigen(PSA) Test NCI Web Page; Gulati et al. 2013; Höti et al. 2023).

However, many early-stage diseases involve biomarkers present at much lower concentrations, often in the picomolar or nanomolar range, making conventional diagnostic methods insufficient. In terms of prostate cancer, miRNA-145, 148, 185 (Coradduzza et al. 2022b) and miR‑146a‑5p, miR‑24‑3p, miR‑93‑5p (Zhang et al. 2021) have been highlighted as diagnostic markers for implementation in the near future. Fluorescence bionanosensors provide an advantage in these cases by offering ultra-sensitive detection, surpassing the limitations of standard biomarker concentration analysis. The aim of the present study is to develop a system suitable for detecting picomolar concentrations of molecules by conventional fluorescence equipment, reliably detecting nanomolar concentrations of fluorophores with a high fluorescence yield.

## Materials and methods

### Materials

All chemicals and reagents used in the experiments were of analytical grade, ensuring precision and accuracy in the results. The study was designed to implement methods that could be quickly deployed and scaled up for future use, aligning with the goal of developing practical diagnostic tools for clinical or research settings.

The reagents included biotin (B4501, Sigma-Aldrich, CAS: 58–85-5) and biotin-fluorescein (Pierce™ Biotin-Fluorescein Conjugate, ThermoFisher Scientific™, CAS: 2420–94-2), both commonly used in biological applications for fluorescence-based detection methods. Additionally, the research incorporated phosphate-buffered saline (PBS) with Tween, a widely used buffer system containing a surfactant. This combination is particularly effective in washing and diluting samples while minimizing non-specific binding, thus enhancing the accuracy and sensitivity of the detection method. In this study, MilliQ water was used to prepare all aqueous solutions, ensuring high purity and reliability (Milli-Q® IQ 7005 Water Purification System, Millipore, Billerica, MA, USA).

Three main components were used for PSA detection. For completing the semi-processed membrane—Human PSA-total ELISA Kit, i.e., anti-Human PSA-total component (Cat. No. RAB0331, UNSPSC Code: 41,116,158, NACRES: NA.32). Human Prostate-Specific Antigen (PSA) was obtained from Sigma-Aldrich (Cat. No. P3235, MDL number: MFCD00165544, UNSPSC Code: 12,352,202), and FITC (Cat. No. 46950, CAS Number: 27072–45-3) was used for part of PSA labeling to apply it as a model analyte. For testing models preparation and membrane testing, both native PSA and FITC-labeled PSA (PSA-FITC) were prepared. Labeling was performed using Fluorescein 5(6)-isothiocyanate (FITC), which binds covalently to amine groups on the PSA molecule, allowing fluorescence-based detection.

For urine-models sample processing and PSA membrane capture, sterile syringes (5 mL and 3 mL, B. Braun Omnifix), flexible tubing, filtration membranes with defined functional concentration, 3D-printed membrane inserts, phosphate-buffered saline (PBS), and sterile collection containers and transport containers were used.

The selection of methods also considered the behavior of the analyte or detection substances, aiming for optimal interaction and binding with sensor materials. Focusing on scalable, easy-to-implement methods and using high-grade reagents and PBS with Tween.

### Methods

#### Preparation and functionalization of nanofibers

**Fig. 1:**
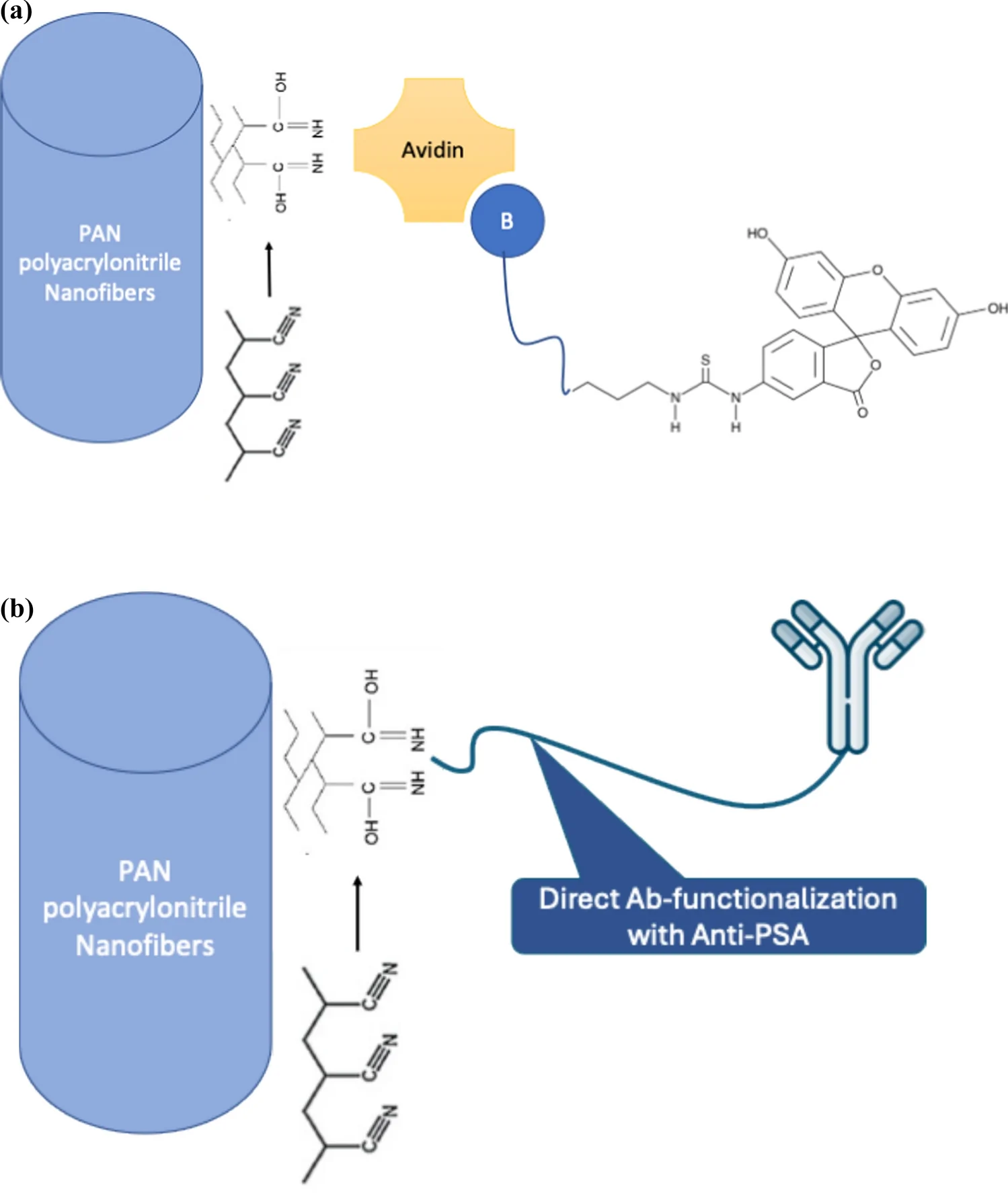
(**a**) Schematic design of the functionalized material. (**b**) Schematic design of the functionalized material with direct Ab-functionalization

The methodology for preparing polyacrylonitrile (PAN) nanofibers began with selecting the PAN polymer for its excellent flexibility, water insolubility, reproducibility in production, and electrospinning into nanofiber mesh. PAN was sourced from ProNanoTech, s.r.o (Czech Republic). Post-production handling, including the surface modification—reduction, aimed to obtain suitable functional groups (− NH), which act as chemical bonds. The nanomaterial functionalization, avidin-linking processing, and direct-antibody functionalization were conducted by EBAS Nano s.r.o. (Czech Republic). The design of the functional material is represented in Fig. 1a.

There are two possible methods of membrane completion. One is completing an avidin-functionalized membrane with a biotinylated antibody, applying avidin as a binding system. The second is direct antibody functionalization of the surface-modified membrane.

The PAN nanofiber surface was chemically modified, typically with amino groups, before membrane functionalization with an avidin linker. This functionalized nanofiber membrane is ready for a highly specific binding of fluorescently labeled biotin. As presented, the semi-ready product could be completed with biotinylated antibodies accessible from the market.

In terms of PSA proof of concept detection, the method of direct Ab-functionalization was employed, in which surface-modified material was directly functionalized with an antibody, which allows quantitative validation. The Human PSA-total ELISA Kit, i.e., anti-Human PSA-total component (Thermo Fisher Scientific), from the material was used for membrane completion. This kit enables sensitive and specific measurement of total PSA levels in serum, plasma, urine, and cell culture supernatants via enzyme-linked immunosorbent assay. PSA and PSA-FITC in body fluids related concentrations has been determined by fluorescence measurement. For the clinical application a filtration protocol for urine samples was developed (represented in methodology section—2.2.5).

PAN nanofiber surface was physically modified and chemically functionalized with antibody, in our experimental setup – Anti-PSA antibody. This setup is ready for binding PSA directly from body fluids.

#### Visualization of nanofibers and diameter determination

Scanning electron microscopy (SEM) was used to visualize the structure of the PAN nanofiber membrane for filtration experiments, utilizing the Vega3 SB SEM (TESCAN a.s. Brno, Czech Republic). Before visualization, samples were coated with a conductive 10 ± 2 nm layer of gold nanoparticles to prevent charging during SEM analysis, using Sputter Coater Q150R (Quorum Technologies Ltd, Lewes, UK). Images were analyzed with ImageJ software (NIH, Bethesda, USA) across five different areas of each sample. Nanofiber diameter distribution was automatically generated by OriginPro software based on manual measurements from SEM images using ImageJ software (Schneider et al. 2012) (“Origin OriginLab Corporation” 2003). Measurements were performed on five different SEM images from distinct areas of the PAN nanofiber sample. Imaging was performed with a VEGA 3 SBU electron microscope (Tescan, Brno, Czech Republic) at magnifications ranging from 500 × to 5000 × . A secondary electron (SE) detector and an accelerating voltage of 10 kV were employed to capture detailed surface morphology.

Multiple SEM images were captured at different magnifications to ensure a statistically significant dataset for diameter estimation. The software’s measurement tools were employed to assess fiber uniformity, distribution, and potential variations in thickness. To enhance accuracy, at least 100 individual fibers were measured across multiple regions of the samples, minimizing localized deviations and providing a more representative average diameter value.

This approach enables a comprehensive assessment of nanofiber morphology, which is crucial for optimizing the electrospinning process and tailoring fiber properties for specific applications, such as biomedical scaffolds, filtration membranes, and biosensing platforms.

#### Filtration setup

**Fig. 2:**
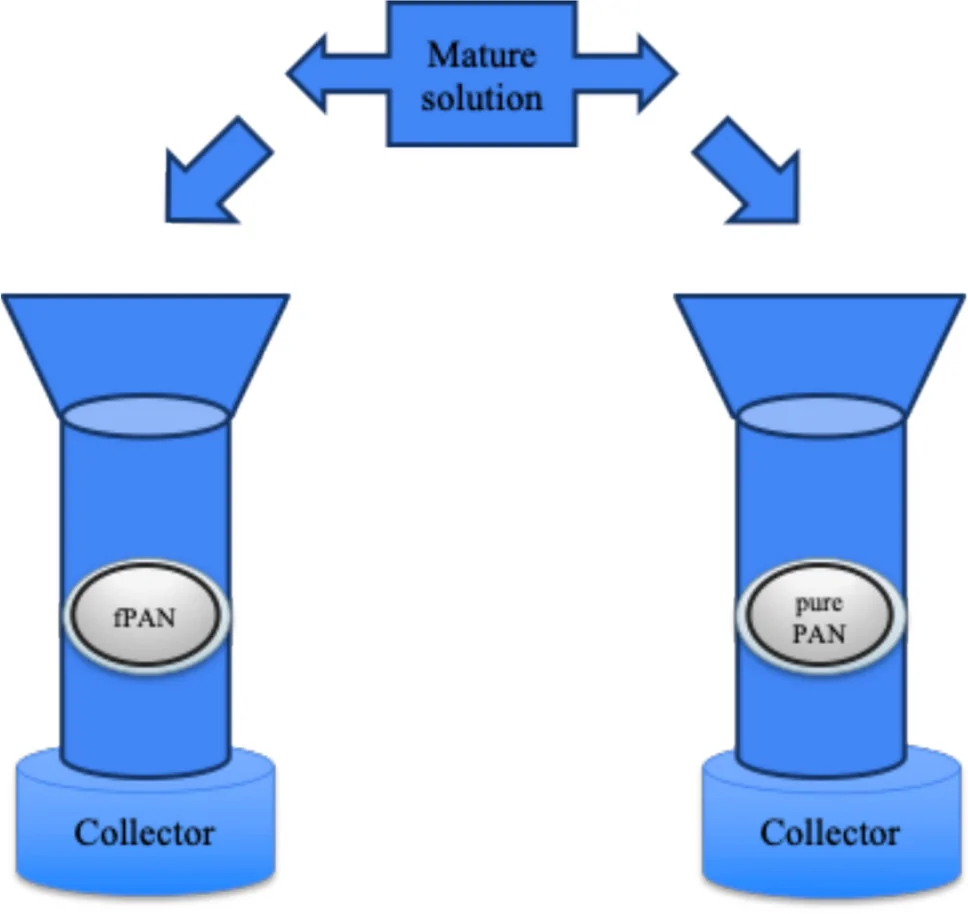
Filtration setup. Specific binding has been determined as a difference between binding on functionalized (fPAN) and non-functionalized (pure PAN) membranes

A filtration setup was designed for PAN nanofiber-based membranes using 3D-printed custom-printed holders. The holders were specifically tailored to fit plastic tubes to hold membranes at the same distance and let all volumes of the analyzed solution pass the membrane. The membranes were positioned securely within the holders and integrated into the plastic tubes. These tubes were connected to a collector for analyte collection Fig. 2.

The solution containing the model biomarker(biotin-FITC) passed through the membrane under the influence of gravity alone without applying any external force. The same setup was used to test both unmodified and functionalized membranes (fPAN). The stock solution was analyzed both before filtration and immediately after passing through each membrane type to evaluate retention and functionality. This approach was also applied to multi-step filtration tests to assess the effectiveness of washing out non-specific binding. These tests helped ensure the reliability and specificity of the functionalized membranes in detecting biomarkers at low concentrations.

#### Body fluid—model PSA detection workflow (filtration-based protocol)

Before sample preparation, all necessary materials, including sterile syringes, tubing, filtration membranes, and phosphate-buffered saline (PBS), were meticulously prepared under sterile conditions. The expiration dates of all membranes and filters were meticulously verified, and PBS solution was pre-aliquoted into labeled transport tubes for post-filtration storage of membranes. Each transport tube was meticulously labeled for traceability with a sample ID and date.

Sample Preparation/Collection: Eight samples with different PSA antigen concentrations (between 0 and 5 ng/mL) have been prepared. The samples were filtered throughout the nanofiber membrane functionalized with PSA antibody as described in the Methods. Subsequently, they were thoroughly washed after filtration, and fluorescent PSA antibody was added.

Urine-model samples (10 mL) were collected into sterile collection containers, each labeled with the sample’s identification details. Basic clinical parameters, such as pH and osmolality (in the future—for urine-model optimisation tests: leukocytes, erythrocytes, and nitrite presence), were recorded on the transport tube or accompanying documentation, as elevated levels could potentially impact PSA binding or recovery.

Sample Processing – Filtration: The collected urine-model sample was drawn into a 5 mL syringe and connected to a membrane filter via flexible tubing. The initial 5 mL was passed through the filter into a clean collection tube. Subsequently, the filtrate was re-filtered once more to enhance the retention of target molecules. This procedure was then repeated with the remaining 5 mL. It was crucial to maintain uninterrupted membrane flow and avoid pressure-related membrane failure. Filtered waste, such as debris or sediment, was meticulously discarded following biosafety protocols.

Membrane Transport and Storage: Following filtration, PBS was meticulously added into a sterile transport tube using a 3 mL syringe. The membrane was carefully removed from the capsule and immersed in PBS to maintain hydration and preserve the captured analytes. Tubes containing the membranes were meticulously stored at 4 °C and transported for subsequent PSA detection fluorescent analysis. In the sense of membrane post-processing – membranes were placed into inserts, filtered with labeled PSA-FITS, and washed out with the same buffer solution afterwards to eliminate non-specific binding. Thereafter the resulting solution was analysed with the fluorescent method.

#### Fluorescence detection

The Fluoromax4 HJY spectrometer measured the fluorescence intensity $({\mathrm{IF}}(\lambda))$ of the samples. with excitation and emission slits set to 2 nm. The system set the excitation wavelength to 490 nm and recorded emission over a wavelength range of 505 nm to 600 nm. Each measurement took approx. 40 s, and the procedure measured each sample three times to obtain a mean fluorescence intensity value $({\mathrm{IFm}}(\lambda))$. The same procedure measured the fluorescence intensity of the buffer $({\mathrm{IFb}}(\lambda))$. The software exported the collected data during the measurements. The calculation corrected the fluorescence intensities for both the sample $({\mathrm{IFs}}(\lambda))$ and the buffer $({\mathrm{IFb}}(\lambda))$ using the equation:

$${\mathrm{IF}}(\lambda)_{\rm cor}={\mathrm{IF}}(\lambda)\ast {\mathrm{Q}}(\lambda)$$

where ${\text Q}(\lambda)$ represents the detectors quantum sensitivity at the respective wavelength. The analysis subtracted the mature solution background and device calibration from the results for both pure PAN membranes and functionalized fPAN membranes to evaluate their binding properties and sensor functionality. 

To calculate the final corrected fluorescence intensity for the sample $({\mathrm{IFs}}(\lambda))$, the formula subtracted the buffer fluorescence intensity $({\mathrm{IFb}}(\lambda))$ from the mean sample fluorescence intensity $({\mathrm{IFm}}(\lambda))$ and multiplied the result by the detector’s quantum sensitivity:

$${\mathrm{IFs}}(\lambda)=[{\mathrm{IFm}}(\lambda)-{\mathrm{IFb}}(\lambda)]\ast{\mathrm{Q}}(\lambda)$$

The processing software generated the fluorescence data, including graphs and illustrations, using OriginPro software (“Origin” 2003). The Bezier smoothing method in Gnuplot 5.4 displayed and compared fluorescence intensity maximums (Gnuplot: An Interactive Plotting Program 2025).

## Results

### Characterization of fractionalized nanofibers

**Fig. 3:**
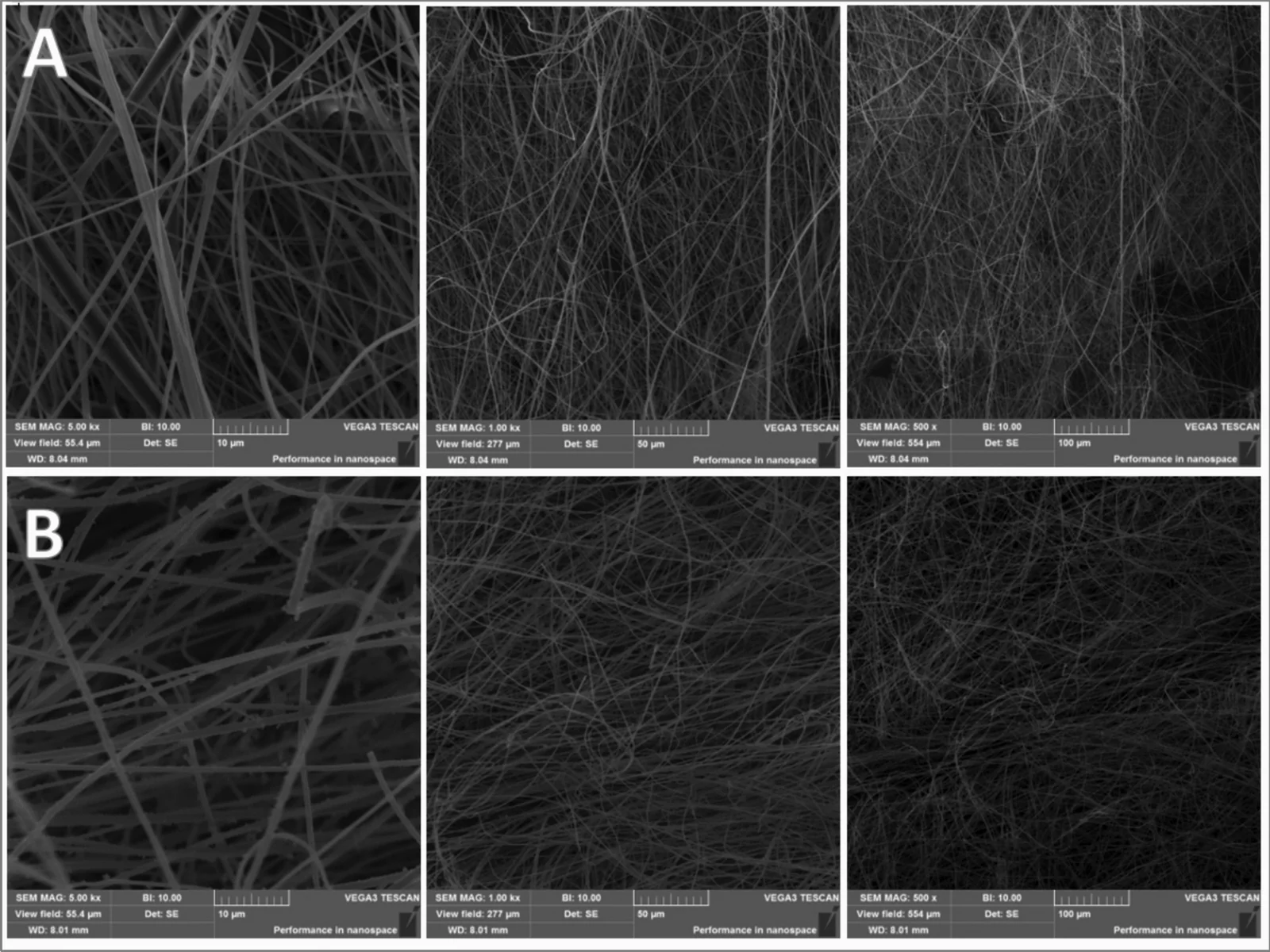
Scanning electron microscopy (SEM) of polyacrylonitrile (PAN) nanofibers. Visualization of nanofiber membranes. The diameter distribution analysis is in Fig. 4. (**A**) Pure PAN (non-functionalized) on Magnification 5000 × , 1000 × , 500 ×. (**B**) fPAN(functionalized) SEM on Magnification 5000 × , 1000 × , 500 ×

Nanofibers were fabricated using electrospinning method from polyacrylonitrile (PAN). A fragment of the PAN nanofiber membrane was preserved as a reference, while the PAN nanofiber surface underwent further processing and functionalization with avidin for the sample preparation, as outlined in the Methods section. The surface morphology of both sample and reference was examined using scanning electron microscopy (Fig. 3). Both samples exhibited a very similar submicroscopic fibrous structure indicating that the functionalization had only a negligible effect on nanofiber membrane morphology.

**Fig. 4:**
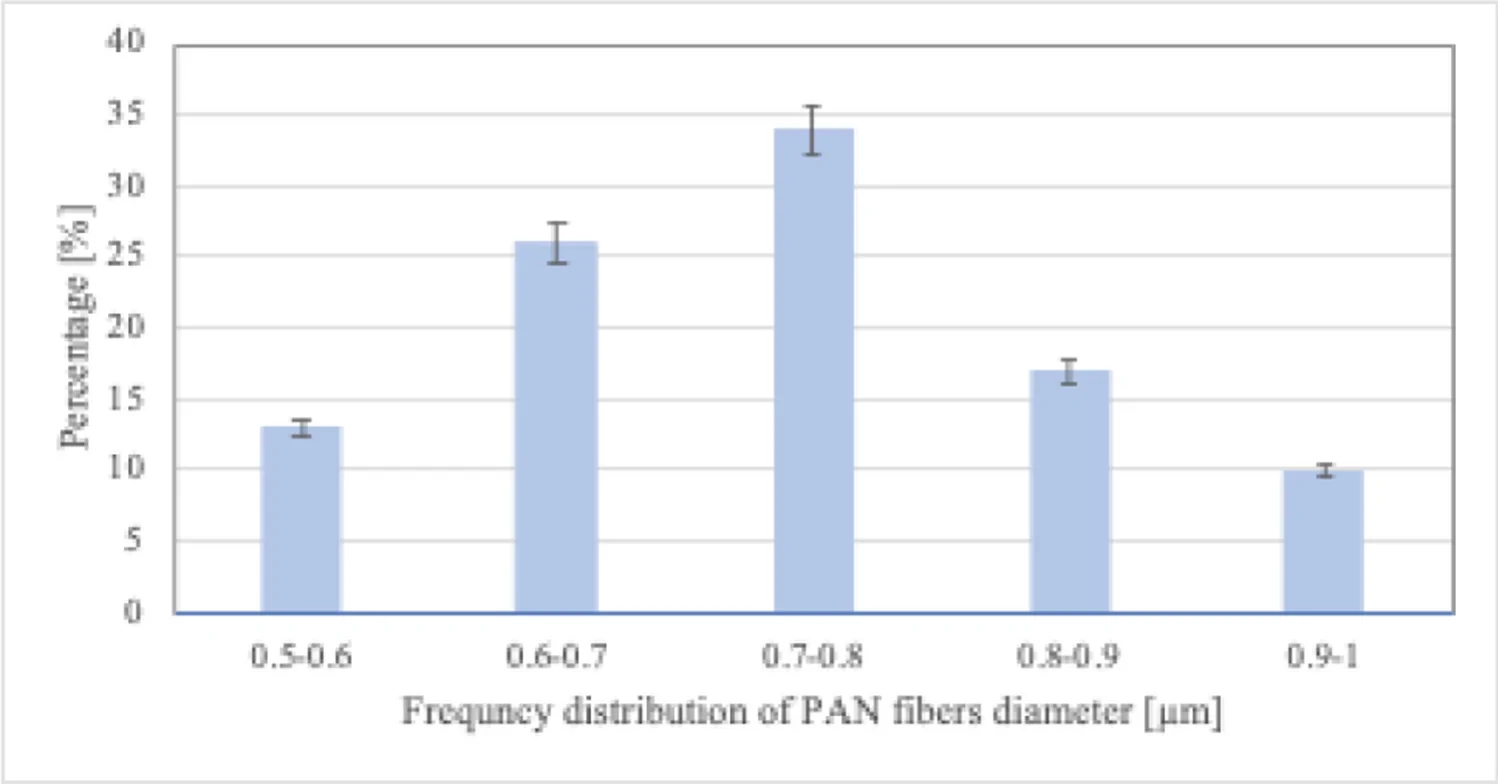
The average diameter of the PAN nanofibers. Diameter distribution analysis based on SEM pictures in Fig. 3. Functionalization has no significant effect on diameter distribution

To quantify the morphological structure, the diameters of at least 50 individual fibers were measured from each image, and the data were analyzed to characterize the structural properties of the nanofiber mesh. The average diameter of the polyacrylonitrile (PAN) nanofibers was determined through detailed analysis of SEM images. As Fig. 4 shows, more than 95% of fiber diameters fall within the interval between 400 nm and 1 μm, creating an optimal structural characteristic for filtration with low obstacles for medical applications. Clearly, the diameter distribution provides key information for the including of the potential biosensor among nanomaterials from a medical device perspective. Consequently, the functionalized PAN nanofiber membrane is an optimal system with a high retention due to a small nanofiber diameter with a high membrane porosity, but still sufficiently well above the diameter of 100 nm fiber size decisive for insertion of the potential biosensor among nanomaterials.

### The sensitivity test

Linearly dependent fluorescence intensity on concentration of fluorophores with a higher quantum yield which is well detected by most fluorometers, is usually reliably observed at the nanomolar concentration range. However, biomarkers in urine and other body fluids typically exist in much lower than nanomolar concentrations. To overcome this obstacle, nanofiber filters can be used to specifically retain biomarkers and measure them at lower volumes, where they become detectable at higher concentrations. This tool helps to prepare a secondary (more concentrated) solution in a lower volume. Clearly, knowledge of the trustworthy concentration range for fluorescence measurement is a key step towards construction of bionanosensors and it is essential for preparation and optimization of nanofiber membrane for application in microfluidic sensors.

**Fig. 5:**
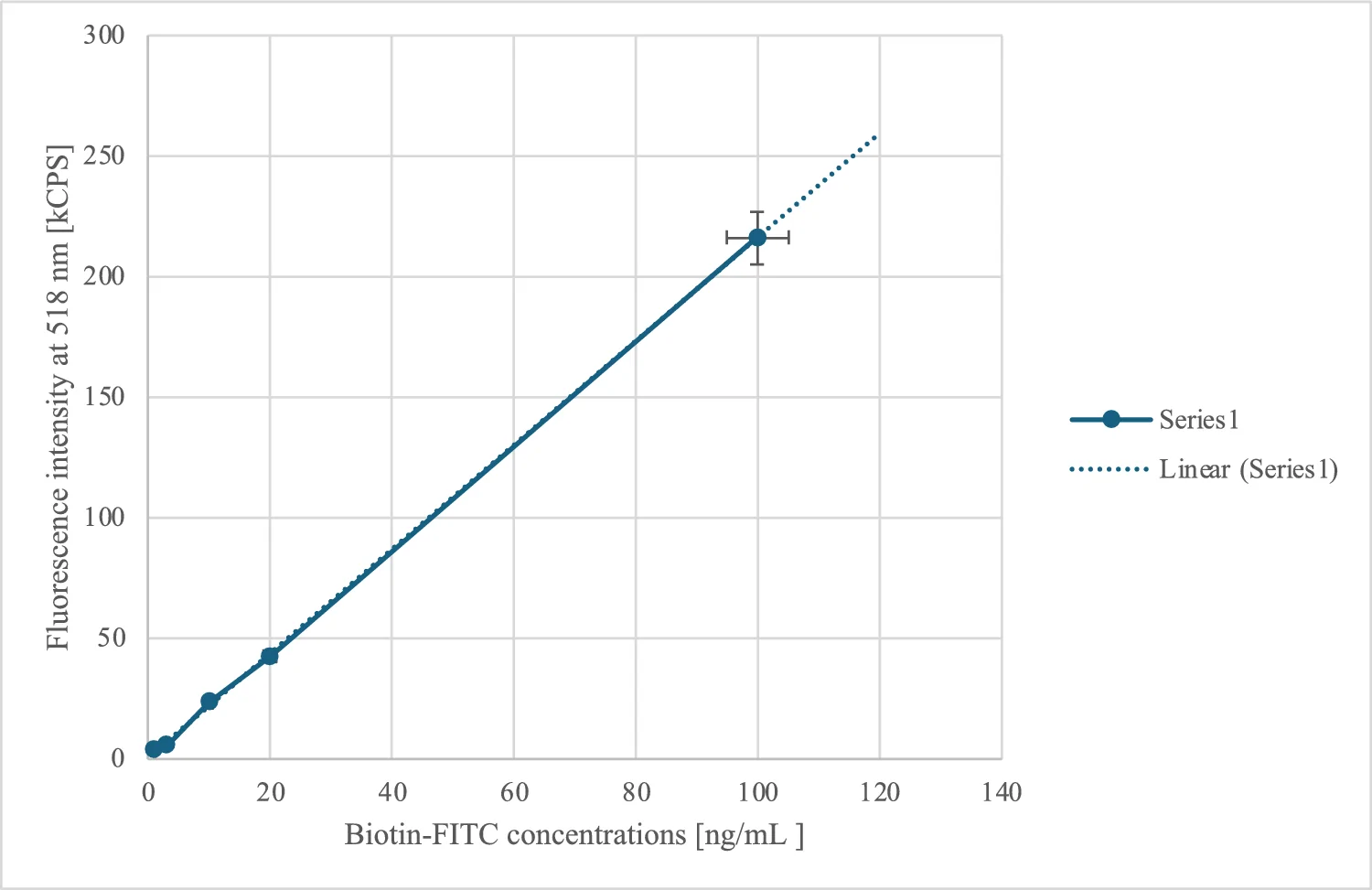
Linear dependence of fluorescent intensity on Biotin-FITC concentration. Fluorescent intensity has been measured at λ = 518 nm (λ_ex_ = 490 nm). kCPS – kilo counts per second

The sensitivity test of the functionalized PAN nanofiber membrane was performed with a model fluid with a fluorescein moiety. A solution of 10 μM biotin-FITC in PBS-Tween was prepared and titrated, then fluorescence intensity was measured as described in the Methods. As shown in Fig. 5, the fluorescence intensity increased linearly throughout the whole titration starting already at the lowest concentration measured (1 ng/mL). This indicates a sufficient sensitivity of the method for the determination of biomarkers of the highest interest, which is supposed to be around dozens of nanomoles and hundreds of picomoles in non-pathological states. This indicates sufficient sensitivity of the method for determination of several of the most important biomarkers (see below in Discussion).

The Biotin-FITC molar mass is 732.80 g/mol), thus, the concentration of 1 ng/mL corresponds to 1.4 × 10⁻⁹ M and 2.5 ng/mL to 3.41 × 10⁻⁹ M. Clearly, fluorescence intensity increased linearly with concentration within this nanomolar range, just relevantly for determination of prostate-specific antigen (PSA) of healthy men.

### Binding capacity of functionalized PAN membranes

**Fig. 6:**
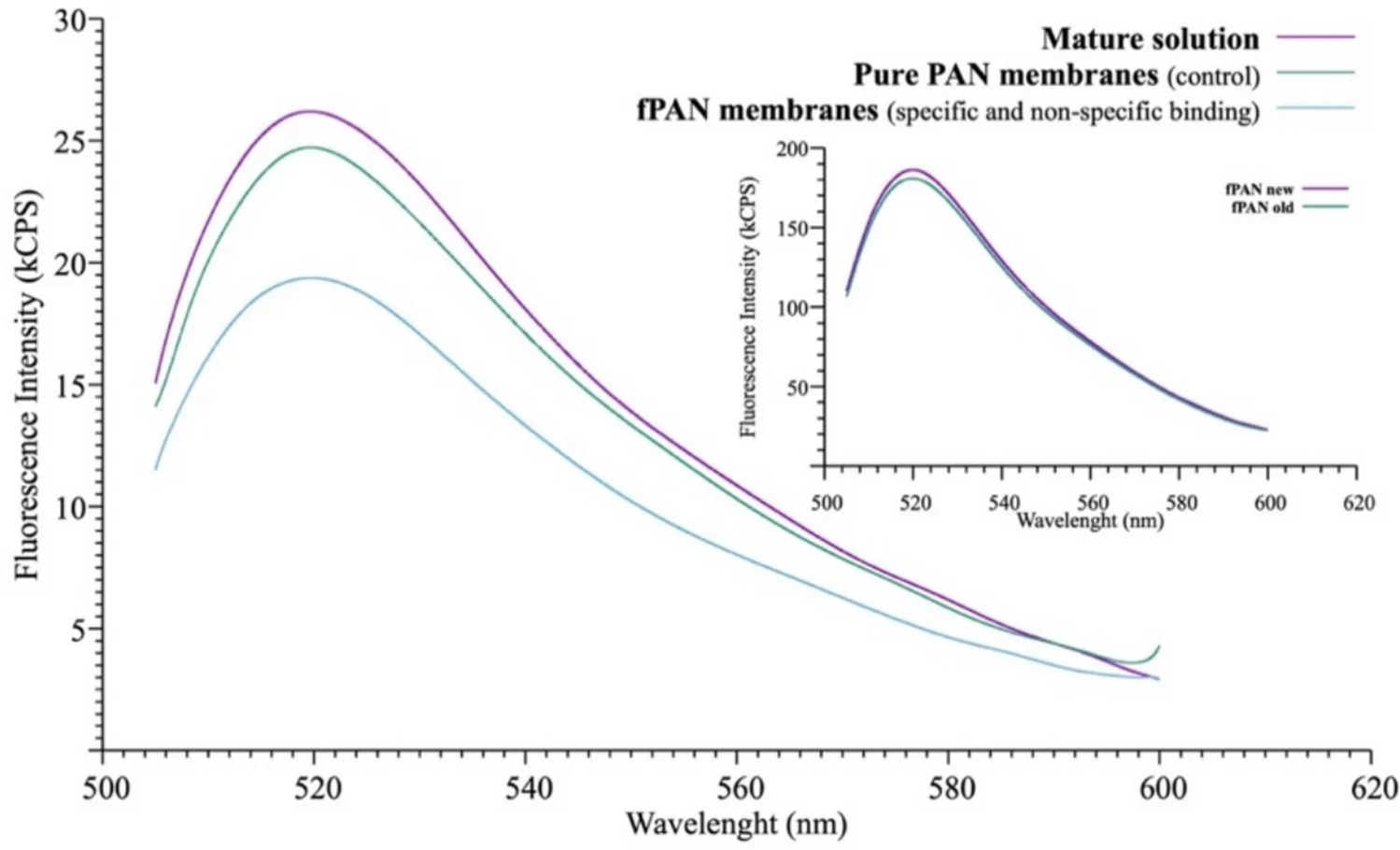
Fluorescence emission spectra of Biotin-FITC bound to PAN nanofibers

After proving the fluorescence detection system as sufficiently sensitive, the next step was determining and optimizing the specific binding capacity of bionanosensors, another key step toward developing nanosensors. To observe the specific binding and also a difference between the specific and non-specific binding, filtration of Biotin-FITC throughout the PAN nanofiber membrane with and without functionalization by avidin, respectively, was performed and compared with the fluorescence of the stock solution (without any filtration). The fluorescence spectra of the FITC-biotin solution were first measured before filtration (purple line in Fig. 6). This solution was, then, filtered through the PAN nanofiber membrane with and without the avidin modification, respectively, and the fluorescence spectra of the filtrate were collected for both samples (green and blue lines, respectively, Fig. 6).

The fluorescence analysis of the functionalized PAN (fPAN) membranes revealed a maximum fluorescence intensity at around λ = 518 nm, which corresponds well with the literature values for the fluorescence of biotin and biotin-fluorescein. Naturally, fluorescence intensity decreased after filtration as a reflection of non-specific and total (specific and non-specific) binding. As Fig. 6 clearly demonstrates, there were significant differences among all three samples. For the pure PAN membranes, the absence of avidin on the membrane surface allowed only non-specific interactions, while the surface-bound avidin caused a stronger binding since besides non-specific binding, significant specific binding was detected.

The results indicate that the selected membrane functionalization efficiently captured the biomarker, which demonstrated the fPAN membranes’ sufficiently high sensitivity for low-concentrated biomarkers. Notably, the surface modification by avidin was also found to be optimal for reliable resolution between specific and non-specific binding.

#### Spectra were taken after filtration through the membrane.

The green spectrum of biotin FITC represents non-specific binding, which is significantly higher than specific binding (blue line). The purple line represents a control sample that has not been filtered. Fluorescent intensity has been measured at λ = 518 nm (λex = 490 nm), kCPS – kilo counts per second.

The functionalized membranes were also tested on their shelf life. The fluorescence intensity was measured after three months of storage. The inset of Fig. 6 clearly shows that pure PAN and fPAN membranes, after filtration, reveal that membrane maturation does not significantly impact the binding capacity. This indicates that once the functionalization process has been completed, the sensor maintains its high level of specificity and efficiency over at least three months. However, future studies should explore the longer-term stability of the functionalized PAN membranes to ensure their reliability in real-world applications.

To test the efficiency of filtration, multi-filtration experiments were performed to observe how many times filtration should be repeated to reach saturation with fPAN membranes.

**Fig. 7:**
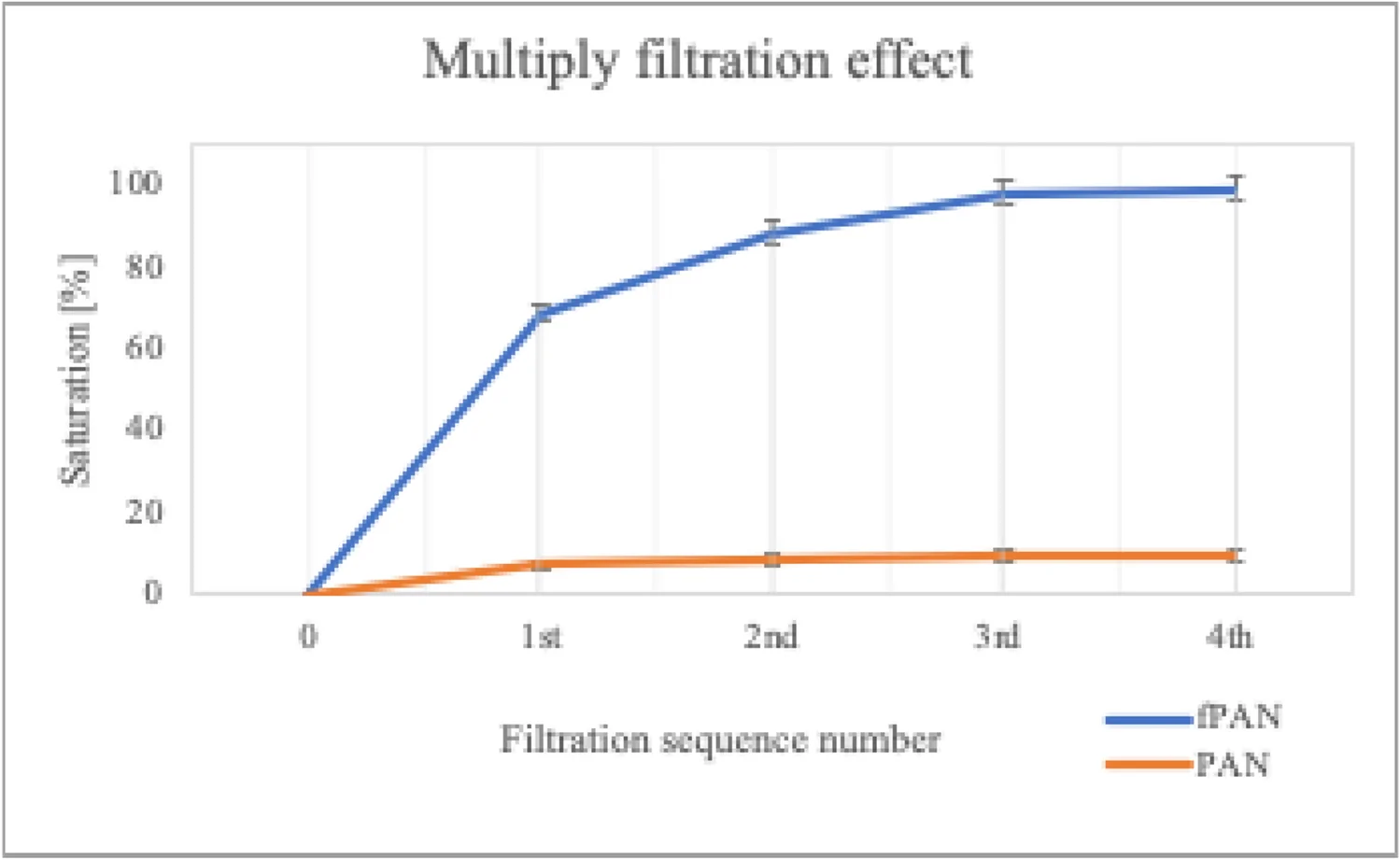
Dependence of saturation capacity on filtration sequence number

Filtration through fPAN membranes was repeated, demonstrating that more than 99% saturation of the membrane’s specific binding capacity occurred after the third re-filtration (Fig. 7). However, the results suggest that while the single re-filtration could serve for a quick orientation test, two filtrations were already sufficient for reliable results.

Our fluorescence analysis and multi-filtration experiments highlight the strong specific binding affinity of functionalized PAN membranes for biotin-fluorescein and suggest that these sensors could be promising for early disease detection and biomarker monitoring.

### Experimental verification of the detection system

**Fig. 8:**
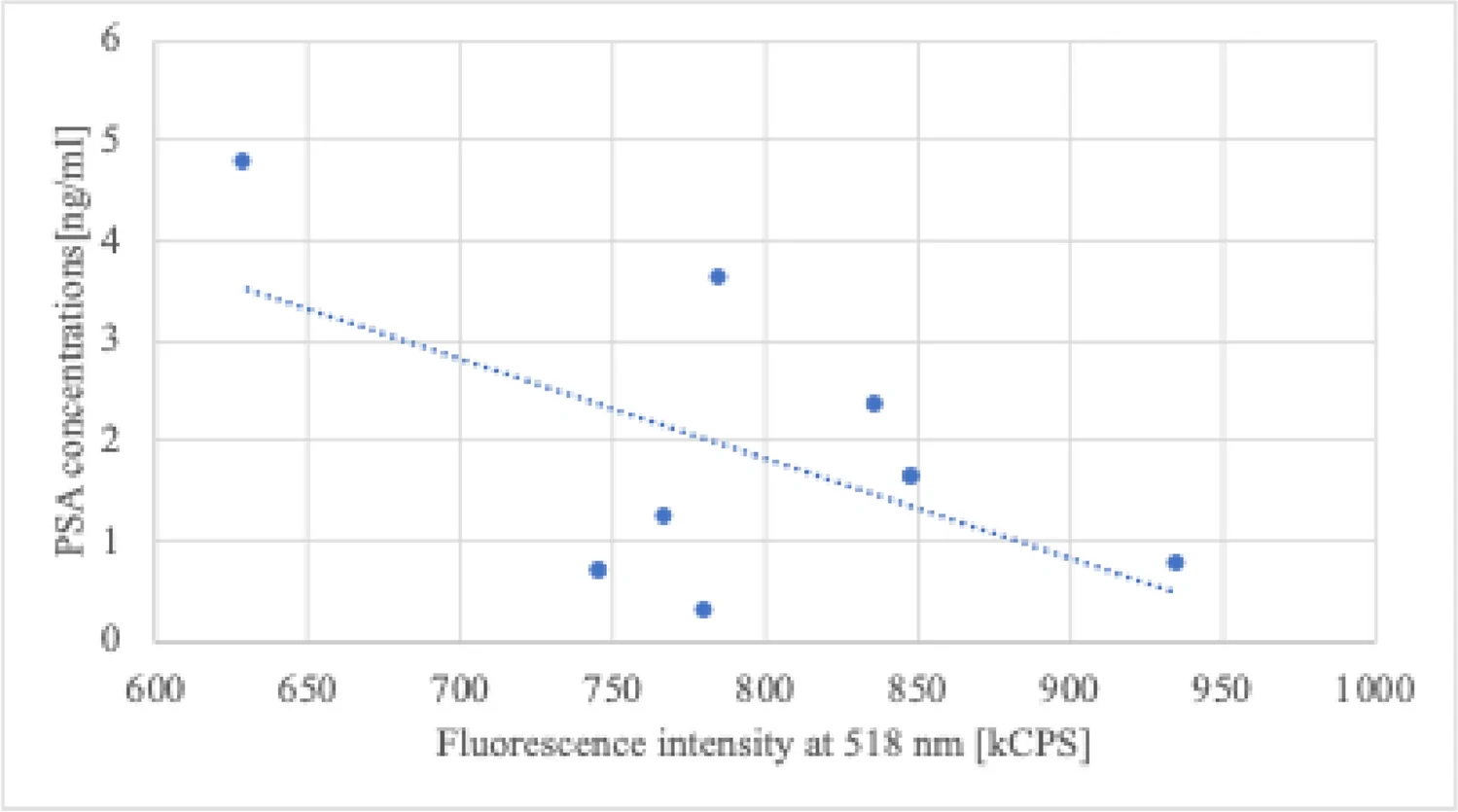
Comparison of PSA concentration determined from model fluids

Experimental verification was performed by fluorescence measurement of our functionalized nanofiber membrane with an antibody against PSA. The concentration of the bound antigen was determined by comparing PSA-antibody fluorescence between the control sample (no PSA filtered) and a respective filtered sample (Fig. 8).

PSA-concentration in mature solution on the axis Y represents sample–concentration of PSA in ng/ml. The axis X shows the final fluorescence of the post-filtrated model fluid. A significant decrease of fluorescence intensity has been observed. This clearly suggests that our system has the capacity to serve as a sensor for the quick determination of markers in human fluids with low scattering. Fluorescent intensity has been measured at λ = 518 nm (λ_ex_ = 490 nm). CPS – counts per second.

## Discussion

This study demonstrates the potential of fluorescence bionanosensors, specifically those based on polyacrylonitrile (PAN) electrospun nanofibers, to detect low-concentration diagnostic biomarkers. Multi-filtration experiments identified a saturation point in the specific binding capacity of fPAN membranes, highlighting their efficiency for low-concentration biomarker detection (picomolar). Functionalization remained stable over time, suggesting long-term sensor reliability. Since urine samples of up to 100 mL can be easily collected from a patient while fluorescence detection requires only 1 mL, the nanofiber system enhances the detection limit by approximately two orders of magnitude. This improvement ensures a reliably measurable fluorescence concentration, reinforcing its suitability for clinical diagnostics.

Both ways of membrane functionalization have their application limitations, such as representative concentration range, in case of direct Ab-attachment, and so the representative aim concentrations should be defined at the beginning and should be verified for each lot. In the case of PAN-Avidin membranes, maximal Ab-concentrations can be adjusted by comparing the filtration of biotinylated antibody and pure biotin. Both methods seem suitable and promising for specific applications, and in their future development, they seem promising in the sense of increasing the sensitivity and accessibility of already developed detection systems. However, there are a few points of improvement in the setups. During the experimental process, several issues were encountered that could compromise the accuracy and reliability of PSA detection in urine samples. One of the primary challenges involved mechanical handling—specifically, difficulties securing nanofiber discs within the membrane holders. Improper fixation could lead to leakage or poor contact between the membrane and the sample, resulting in inconsistent sample flow or insufficient analyte capture. This issue may also contribute to uneven antibody distribution, affecting signal uniformity. Custom-designed 3D-printed holders with precise dimensions and integrated sealing features can be employed to resolve these challenges. These supports would help stabilize the disc, maintain even pressure distribution, and prevent leakage.

Another concern was related to fluorescence signal acquisition. Variability in fluorescence intensity may have resulted from uneven sample distribution, inconsistent antibody coverage, or misaligned detection optics. To minimize these issues, rigorous standardization of the calibration curve preparation is essential, using serial dilutions with replicates to ensure a robust dynamic range. Furthermore, optimizing imaging settings and ensuring consistent sample placement in the detection setup can reduce signal variability. By addressing these sources of variability, the sensitivity and reproducibility of PSA detection using the developed nanofiber-based biosensor platform can be significantly improved.

However, the receptor in a stage of the semi-product should be optimized for certain applications. In the case of the detection of biomarkers with an fPAN bioreceptor, there are a few essential parameters that should be considered: diagnostically important concentration range, defined by cut-off (or correlation to classification of disease diagnostic algorithms, in a future stage of development of quantitative analysis). This parameter could be reflected at the stage of membrane diagnostic setup preparation in the following factors: size of the membrane, time of interaction, defined all spots full saturation (reference membrane) and volume of samples.

The affinity of the binding spots on the final bioreceptors is an essential factor for smooth transfer from laboratory conditions to experimental model fluids and even clinical testing. The affinity will be defined by the affinity of the used biotinylated Ab, which will interact with the antigen and the labeled antigen. Surely, some factors should be taken into consideration, as homogeneity of the avidin spots and Ab-Ab interactions, which should be defined at the advanced stage.

Early disease detection is crucial for improving patient outcomes, particularly when timely intervention enhances survival rates, such as cancer and infections. Among the biomarkers of high interest for simple urine-based detection, PSA remains one of the most established (Jain et al. 2023). It serves as a key biomarker for prostate cancer diagnostics, with clinically significant concentrations typically measured in the nanomolar to picomolar ranges (Cimmino et al. 2021; David and Leslie 2024). Serum PSA levels are already included in screening algorithms in many countries (Tidd-Johnson et al. 2022; Zhang et al. 2016). However, urinary PSA detection presents challenges due to influencing factors and low specificity in urine-based tests. Benign prostatic hyperplasia (BPH)—a non-cancerous enlargement of the prostate gland—and prostatitis, an inflammation or infection of the prostate, can affect PSA levels. Additionally, recent ejaculation and physical activities, such as cycling, can temporarily elevate PSA levels (Prostate-Specific Antigen (PSA) Test—NCI). Due to these factors, many studies have established a higher standard cut-off concentration for urinary PSA detection, commonly reported as 150 ng/mL (Bolduc et al. 2007). A more recent study has demonstrated the corresponding range, and in research referred to as aggressive prostate cancer detection, it concluded that urinary PSA could complement serum PSA and serve as a surrogate biomarker for predicting aggressive prostate cancer (Höti et al. 2023). Given PSA’s molar mass (28,430 g/mol), this corresponds to a molar concentration of approximately 5.27 nM, which aligns well with the detection capabilities of fluorescence-based biosensors, such as the Biotin-FITC system used in this study.

Besides serum PSA-based screening and non-specific urinary PSA detection, urine-based diagnostic tests are already available on the market. These tests measure microRNAs and other markers in urine or urine Precipitate —such as PCA3 mRNA, Annexin A3, DLX1, HOXC6, TMPRSS2-ERG gene fusion, and PSA in combination with the MPS2 test —which could help prevent unnecessary biopsies in individuals with elevated serum PSA levels (Salagierski and Schalken 2010). Additionally, tests like ExoDx, which analyzes exosomal PCA3, SPDEF, and ERG RNA, offer non-invasive screening methods for prostate cancer. Other assays, such as those analyzing small non-coding RNAs like the Sentinel PCa Test, also show promise in the early detection of prostate cancer (Raja et al. 2018). The present research aims to develop a system that can be customized with spread variations of biotinylated antibodies, including diagnosing higher concentrated bacteria to lower concentrated traces of proteins (including Collagen I urine loss in osteoporosis, or Collagen II, and a correlated set of markers, to describe degenerative musculoskeletal diseases molecularly. Future developments in the technology include SERS (Surface-Enhanced Raman Spectroscopy) analysis of the receptors specifically developed to detect lower concentrations and even miRNA, at the moment in experimental-laboratory conditions (Martino et al. 2024, 2025).

The method of using an fPAN membrane depends on its method of analysis and practical application. In terms of clinical application, several factors seem to be most important, including developing the ¨New-generation-receptors ¨ for “New-generation-biomarkers” and the transfer of advanced laboratory techniques to the bedside. As well as well-described microRNAs, acting as biomarkers, or even targeted markers for various pathological states, including prostate cancer (Coradduzza et al. 2022b,a; Pashchenko et al. 2023).

The functionalized PAN membranes demonstrated strong specific affinity to the biomarker, while non-functionalized PAN membranes showed a minimal interaction. This finding enables rapid and straightforward detection of low-concentration biomarkers and, thus, diseases at their early stage, as well as monitoring of healing progress from body fluids directly in medical contexts. The use of urine as a diagnostic medium is a significant focus in modern personalized medicine since it is crucial for prompt interventions and enhancing clinical decision-making accuracy. Integrating these sensors into portable devices would offer a practical and cost-effective solution for point-of-care diagnostics (Yang et al. 2022; Yammouri, Ait Lahcen 2024, Min et al. 2021, Tasoglu et al. 2022).

Further exploration and testing of real urine for biomarker interactions with functionalized nanofibers is crucial for real-world applications. The platform’s adaptability for multi-marker assessments and easy functionalization using biotinylated antibodies enhances its clinical utility. As seen in previous studies, such as those by (Nan et al. 2023) who explored biosensor sensitivity in disease detection, the effectiveness of fluorescence-based sensors is increasingly being recognized in early diagnostics (Lai et al. 2012). Moreover, advances in the sensitivity and specificity of these sensors have been shown to improve early detection capabilities, as supported by studies in optical sensing (Kumar et al. 2024).

The analysis of the amount of bound biomarker to the membrane initially involved two methods: electrical detection of impedance changes, suitable for higher concentrations, and a fluorescent method (Prokůpek et al. 2024). So, labeling the detecting marker is essential at this stage of development. However, there are still available ways of implementing the analysis. Firstly, the fluorescence analysis could be improved by directly analyzing the surface fluorescence of the membrane. Secondly, fluorescent-labeled marker could be added to the sample as a competitive marker in a known concentration to eliminate secondary filtration. Furthermore, the labeling of biomarkers is not limited to standard labeling molecules; nowadays, advanced standards are achieving methods of development, such as nanodots and carbon/quantum dots. So, for specific applications, an optimal setup should be found, if PSA labeling is appropriate, depending on achieved results. However, the method of membrane analysis shows the importance of stratification of the diagnostic pathway application point. For pre-screening, bacteria, semi-quantitative, qualitative, and electrical analysis of the membrane could be enough. For screening and diagnosing within different types of biomarkers, optical/fluorescent methods will be suitable. Future applications, such as novel miRNA and miniaturizing genomic analysis, using the SERS method could fulfill the goal.

## Conclusion

This study developed a fluorescence bionanosensor based on functionalized polyacrylonitrile (fPAN) nanofibers, which is sufficiently sensitive and selective for detecting biomarkers in fluids. The study showed both the detection specificity and sensitivity of the system in the picomolar range. This biomarker concentration is significant for early-stage disease detection in urine. Bionanosensors showed their potential for a direct detection from human fluids, namely urine, but also for their involvement in microfluidic systems (Su et al. 2022). The idea of using electrospun nanofibers as bioreceptors includes connection and adjustments/ customisation and optimization of the whole process of biosensor development.

In conclusion, the development of PAN nanofiber-based optical fluorescence bionanosensors presents significant potential for enhancing the sensitivity and specificity of early-stage disease detection, as well as its various clinical, environmental, and veterinary applications (Amler et al. 2024; Bocková et al. 2022; Huang et al. 2024; Serra et al. 2024; Pashchenko et al. 2024). By refining the functionalization processes and optimizing sensor parameters, these technologies are likely to show the way for more accurate, rapid, and accessible diagnostic tools, ultimately contributing to improved patient outcomes and more effective healthcare strategies (Pashchenko et al. 2022).

## Data Availability

Please contact the primary author for data requests.

## Abbreviations

AVID: Avidin
AUC: Area under the curve
BEST: Biomarkers, endpoints, and other Tools
BPH: Benign prostatic hyperplasia
Biotin-FITC: Biotin-fluorescein conjugate
ExoDx: Exosomal RNA-based prostate cancer diagnostic test
fPAN: Functionalized polyacrylonitrile
FITC: Fluorescein isothiocyanate
IF: Fluorescence intensity
kCPS: Kilo counts
MilliQ: High-purity water system
MPS2: Multiparametric prostate cancer screening test
PAN: Polyacrylonitrile
PBS: Phosphate-buffered saline
PCA3: Prostate cancer antigen 3
PDEF: SAM pointed domain-containing ETS transcription factor
PSA: Prostate-specific antigen
Q(λ): Quantum sensitivity of the detector
RFU: Relative fluorescence units
ROC: Receiver operating characteristic
SBU: Secondary electron detector
SEM: Scanning electron microscopy
SERS: Surface-enhanced raman spectroscopy
TMPRSS2-ERG: Transmembrane protease serine 2 – ETS-related gene fusion
VEGA 3 SBU: Scanning electron microscope model from tescan
